# Nanonaringenin and Vitamin E Ameliorate Some Behavioral, Biochemical, and Brain Tissue Alterations Induced by Nicotine in Rats

**DOI:** 10.1155/2021/4411316

**Published:** 2021-09-24

**Authors:** Mohamed A. Kandeil, Eman T. Mohammed, Rania A. Radi, Fatma Khalil, Abdel-Razik H. Abdel-Razik, Mohamed M. Abdel-Daim, Ghada M. Safwat

**Affiliations:** ^1^Department of Biochemistry, Faculty of Veterinary Medicine, Beni Suef University, Beni Suef 62511, Egypt; ^2^Animal and Poultry Management and Wealth Development Department, Faculty of Veterinary Medicine, Beni Suef University, Beni Suef 62511, Egypt; ^3^Department of Histology, Faculty of Veterinary Medicine, Beni Suef University, Beni Suef 62511, Egypt; ^4^Department of Pharmaceutical Sciences, Pharmacy Program, Batterjee Medical College, P.O. Box 6231, Jeddah 21442, Saudi Arabia; ^5^Pharmacology Department, Faculty of Veterinary Medicine, Suez Canal University, Ismailia 41522, Egypt

## Abstract

Nicotine is the major alkaloid present in cigarettes that induces various biochemical and behavioral changes. Nanonaringenin (NNG) and vitamin E are antioxidants that are reported to mitigate serious impairments caused by some toxins and oxidants. Thus, we aimed to investigate the efficacy of NNG, vitamin E, and their combinations to ameliorate behavioral, biochemical, and histological alterations induced by nicotine in rats. Adult male albino rats were randomly grouped into six equal groups (10 rats/group): control, N (nicotine 1 mg/kg b.w./day S/C from 15^th^ to 45^th^ day, 5 days a week), NNG (25 mg/kg b.w./day orally for 45 days), N + NNG, N + E (nicotine + vitamin E 200 mg/kg b.w./day orally), and N + NNG + E (nicotine + NNG + vitamin E at the aforementioned doses). Behavioral tests were conducted on day 15 and 30 postnicotine injection, while memory tests, brain neurotransmitters, antioxidants, and histopathological examination were examined at day 30 only. As a result, nicotine impaired rats' activity (hypoactivity and hyperactivity) and memory, induced anxiolytic and anxiogenic effects on rats, and altered neurotransmitters (acetylcholinesterase, serotonin, and dopamine), and redox markers (MDA, H_2_O_2_, GSH, and catalase) levels in brain homogenates. Thickening and congestion of the meninges and degeneration of the cerebral neurons and glia cells were observed. Cosupplementation with NNG, vitamin E, and their combination with nicotine was beneficial in the alleviation of activity impairments and improved short memory and cognition defects and exploratory behaviors. Our results indicate the antioxidant potential of NNG and vitamin E by modulating redox markers and neurotransmitters in the brain. Thus, data suggest that the prophylactic use of NNG, vitamin E, and/or their combination for (45 days) may have a successful amelioration of the disrupted behavior and cognition and biochemical and histopathological alterations induced by nicotine.

## 1. Introduction

Tobacco smoking caused death to 100 million persons worldwide in the 20^th^ century and is expected to kill about one billion people in the 21^st^ century if the same manners remain [1]. The major alkaloid that existed in cigarettes is nicotine (N) about 1–2 mg/ml and was detected in the smoker's blood [2]. Nicotine activates the nicotinic acetylcholine receptors (nAChRs) present in the brain [3]. The nAChRs activation enhances the liberation of various neurotransmitters such as acetylcholine, glutamate, dopamine (DA), noradrenaline (NDA), and gamma amino butyric acid (GABA) in the brain [4, 5]. These neurotransmitters play an important role in modulating a great number of behaviors, including locomotion, anxiety, exploration, learning, and memory [3]. In addition, nicotine is implicated in the production of free radicals and reactive oxygen species (ROS) and generates oxidative stress [6, 7] which leads to mitochondrial dysfunction that causes neural death [8]. Therefore, antioxidants are highly needed by brain for the high oxygen demands and long-life duration of the neurons [9]. Recently, natural antioxidants have attracted more attention to face free radical damage in different tissues in rats induced by various oxidants [10–14]. Flavonoids are a group of natural products that have valuable biological activities, including antioxidant, anti-inflammation, and antitumor properties [15–17]. Among flavonoids, naringenin (NG) known as 4′,5,7-trihydroxy flavanone, a citrus flavanone, has rapid circulation bioavailability [18]. Naringenin has antioxidant [19], anti-inflammatory [20], and antidepressant effects [21]. In addition, vitamin E is an antioxidant that could compete for oxidative stress status, such as neurodegenerative diseases [22]. Hence, NG and vitamin E could successfully alleviate learning and cognition deficits induced by lipopolysaccharide and aesthesia, neurotoxicity, chronic stress in Alzheimer's disease model and diabetic rats [23–28]. Moreover, NG mitigated anxiety-like effects induced by iron and hypoxic stress in rats [29, 30].

The larger surface area of nanoparticles per mass unit and their smaller size can lead to faster drug delivery and higher bioactivity compared to bulky particles [31–33]. Thus, nanosize form naringenin (NNG) may have more ameliorative action against the serious effects of oxidants than NG. Moreover, little is known about the prophylactic use of nanosize form naringenin (NNG) and vitamin E combination to mitigate behavioral and memory disruption caused by oxidants such as nicotine.

Therefore, we aimed to evaluate the prophylactic role of NNG, vitamin E, and their combination in amelioration of impaired activity, anxiety, and learning induced by nicotine as well as biochemical and histological alterations posed by subcutaneous nicotine administration in rats.

## 2. Materials and Methods

### 2.1. Chemicals

Nicotine hydrogen tartrate salt (C_10_H_14_N_2_) (MW = 462.41 anhydrous 95% nicotine, with CAS number 65-31-6) and naringenin (C_15_H_12_O_5_) (4′,5,7-Trihydroxyflavanone MW = 272.26, purity $95% with CAS Number: 67604-48-2) were purchased from Glentham Life Sciences Ltd., England Unit 5 Ingoldmells Court Edinburgh Way, Corsham Wiltshire SN13 9XN, the United Kingdom. Vitamin E (dl-Alpha Tocopheryl Acetate) (C_29_H_50_O_2_) with item number 1770 was purchased from Puritan's Pride, INC Ronkonkoma, NY 11779, USA. The commercial diagnostic kits used for assaying of the reduced glutathione (GSH), malondialdehyde (MDA), catalase, hydrogen peroxide (H_2_O_2_), and acetylcholinesterase (AchE) were obtained from Biodiagnostic Company for Research Kits, Egypt. Enzyme-linked immunosorbent assay (ELISA) kits for rat dopamine (Catalog Number: MBS725908) and rat 5-hydroxytryptamine (5-HT) (Catalog Number: MBS725497) were provided by R&D System, USA. All other chemicals were of analytical grade.

### 2.2. Nanonaringenin Preparation

A high-energy ball milling technique was used to prepare nanonaringenin according to the method of Gusev and Kurlov [34]. NNG was prepared at Nanotechnology Lab, Faculty of Postgraduate Studies for Advanced Sciences, Beni-Suef University.

### 2.3. NNG Characterization

**Figure 1:**
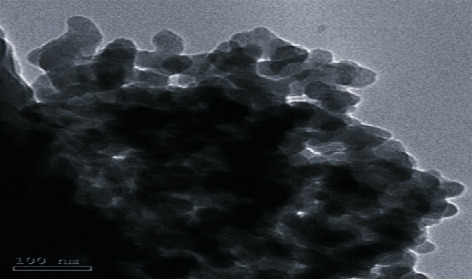
TEM images of NNG showing the shape and size of NNG, which appear as spherical particles with an average size of 100 nm.

TEM electron microscope (Model: JEM-2100, JEOL Ltd., Tokyo, Japan) was used for the characterization of NNG at the National Research Center, Dokki, Giza, Egypt. A droplet of a freshly prepared suspension was poured onto copper grids and left to dry in the air, then observed by high-resolution TEM. The TEM indicated that NNG droplets were nearly spherical crystal shaped with homogeneous nanometric size spreading (Figure 1).

### 2.4. Experimental Design

The present study was carried out on 60 adult male albino rats ranging between 120 and 150 g body weight. They were obtained from Helwan Farm of Laboratory Animals, Cairo, Egypt. Rats were acclimatized for 2 weeks, then they were housed in groups in metal cages under good ventilation and illumination conditions at room temperature (24°C ± 2°C), humidity (68%) under 12 hours light-dark cycle during the period of the experiment. Rats had free access to water and diet ad libitum. All experimental measures were performed in a strict guideline according to the recommendations for the care and use of laboratory animals and approved by the Institutional Animal Care and Use Committee at Beni-Suef University.

The rats were randomized into six equal groups (*n* = 10/group).

#### 2.4.1. Control Group

The rats were injected subcutaneously with 0.2 ml/kg b.w. of 0.9% physiological saline only (vehicle).

#### 2.4.2. Nicotine Group (N)

The rats were injected subcutaneously with freshly prepared nicotine dissolved in physiological saline (1 mg/kg b.w./day) for 30 days (15^th^–45^th^ day) 5 days a week [35]. This dose delivered a plasma nicotine level in rats equal to the quantity absorbed from the smoking of 20 cigarettes by human persons [36]. The LD_50_ of nicotine is 50 mg/kg for rats [37].

#### 2.4.3. Nanonaringenin Group (NNG)

The rats received NNG (25 mg/kg b.w./day) orally with a gastric tube for 45 days [38, 39].

#### 2.4.4. Nicotine + Nanonaringenin Group (N + NNG)

The rats were given NNG (25 mg/kg b.w./day) for 45 days orally with a gastric tube, interrupted by nicotine injected subcutaneously (1 mg/kg b.w.) at the 15^th^–30^th^ day. Nicotine was given an hour before NNG administration.

#### 2.4.5. Nicotine + Vitamin E Group (N + E)

The rats were given vitamin E dissolved in corn oil orally with a gastric tube for 45 days (200 mg/kg b.w./day) [40], interrupted by nicotine injected subcutaneously (1 mg/kg b.w.) at the 15^th^–30^th^ day. Nicotine was given an hour before vitamin E administration.

#### 2.4.6. Nicotine + Nanonaringenin + Vitamin E Group (N + NNG + E)

The rats were given NNG (25 mg/kg b.w./day) and vitamin E (200 mg/kg b.w./day) orally with a gastric tube for 45 days interrupted by nicotine injected subcutaneously (1 mg/kg b.w.) at the 15^th^–30^th^ day.

### 2.5. Behavioral Measurements

The following behavioral tests were performed to evaluate the effect of the nicotine, NNG and/or vitamin E with nicotine injection on rats' activity and memory. Five rats per group were used. The tests were performed two hours after the nicotine injection.

#### 2.5.1. Open Field Test

This test was conducted on days 15 and 30 after injection of nicotine. This test measured locomotion, exploration, and anxiety, according to Gould et al. [41]. The apparatus was constructed according to Brown et al. [42]. The rat was placed into one of the four corners of the open field and permitted to discover the apparatus for 5 minutes. Behavior was analyzed according to Walsh and Cummins [43], Choleris et al. [44], and Kalueff and Tuohimaa [45].

For each rat, locomotion anxiety like behaviors (number of peripheral squares crossed with all four paws and rearing; frequency with which the rat stands against wall of the maze), freezing (immobility) time, exploration (number of center square entries with all four paws and time rats spent in them) were measured.

#### 2.5.2. Learning and Memory Tests

*(1) Y-Maze Test*. This test was conducted on day 30 of the experiment. The test is used to measure spatial short-term working memory by recording the spontaneous alternative behavior in the maze arms [46]. According to the procedure described by Wall et al. [47], Rasoolijazi et al. [48], and Baluchnejadmojarad et al. [49], arms were marked as A, B, and C; then, each rat was placed at the beginning of (A) arm and left for eight minutes and then cleaning maze with 70% alcohol after each rat. Overlapping triplet sets (i.e., ABC CBA ABC) were used to calculate the sequence of arm entries. Spontaneous alternation behavior percentage (SAP) was measured in the Y-maze to evaluate working memory.

(1) $$\begin{array}{c} \text{SAP}=\frac{\text{actual alternation}}{\text{maximum alternation }\left(\left(\text{total arm entries}-2\right)\right.}\times 100. \end{array}$$

*(2) Novel Object Recognition (NOR)*. The aim of this test is to evaluate the short-term memory and was performed at the end of the experiment. Novel object recognition test is used to evaluate learning and memory deficits in rats and mice depending on the natural behavior of a rat in interacting with novel objects more than familiar objects, which are known as recognition memory [50] according to the protocol designated by Bevins and Besheer [50], Leger et al. [51], and Lim et al. [52]. NOR was evaluated by calculation of discrimination index (DI).

(2) $$\begin{array}{c} \text{DI}=\frac{\text{time exploring novel object}}{\text{total time exploring both novel and familiar objects}}. \end{array}$$

### 2.6. Sampling and Tissue Preparations

Twenty-four hours following the last dose, the animals were killed by cervical dislocation. Brain tissues were collected and washed with physiological saline (NaCl 0.9%), then divided into three parts. The first portion was preserved in a neutral buffered formalin solution for histopathological examination. The second portion was homogenized in 10 volume phosphate buffer saline (pH: 7) using a homogenizer (Ortoalresa, Spain), then centrifuged at 20,000 ×g for 15 minutes at 4°C. The supernatant was kept at −80°C for further biochemical assays of GSH, MDA, Catalase, H_2_O_2_ levels, and AchE activities. The third portion of brain tissue was kept at −80°C for ELISA assays of dopamine and serotonin.

### 2.7. Biochemical Assays

The brain homogenate was used for the measurements of MDA, GSH, H_2_O_2_ levels, and catalase activity according to the methods described by Satoh [53], Beutler and Kelly [54], and Aebi [55], respectively. AchE activity was estimated in brain tissue homogenate according to the method of Kovarik et al. [56]. Levels of dopamine and serotonin in rat brain tissues were estimated by competitive enzyme- immunoassay technique (ELISA) [57] using rat ELISA kits for dopamine and rat 5-hydroxytryptamine (5-HT) or serotonin following the manufacturer's instructions.

### 2.8. Histopathological Examination

Brain tissues were dissected carefully and washed with physiological saline (NaCl 0.9%), then immersed in neutral buffered formalin solution 10%. Tissue specimens of the brain were dehydrated in ethyl alcohol, cleared in xylol, impregnated in soft paraffin, and embedded in hard paraffin. Sections of 4–6 *μ*m were cut and mounted on clear and dry glass slides. The obtained slides were stained with Hematoxylin and Eosin (H & E) and Bielschowsky's silver stain for histopathological examination using LEICA (DFC290 HD system digital camera, Heerbrugg, Switzerland) connected to the light microscope using 10, 20, 40 objective lenses [58].

### 2.9. Statistical Analysis

The results were statistically analyzed by using Graph Pad In stat software (version 3, ISS-Rome, Italy). One-way analysis of variance (ANOVA) test followed by Tukey's post hoc test for experimental group comparison was used. The *p* values below 0.05 were accepted for significance.

## 3. Results

### 3.1. Behavioral Measurements

Table 1 shows that nicotine induced a marked decrease and increase in locomotor behaviors of the treated rats. On day 15 after injection of nicotine, the crossed peripheral squares number was decreased significantly (*p* < 0.05) in N and N + NNG groups compared to the control one. At day 30 of nicotine injection, a significant (*p* < 0.05) decrease was observed in N and N + E in comparison to the control group. On the contrary, on day 15, rearing showed a significant (*p* < 0.05) increase in the N group while a significant (*p* < 0.05) decrease was observed in N + NNG, N + E and N + NNG + E compared to the control and nicotine groups. At day 30 of nicotine injection, rearing was significantly (*p* < 0.05) reduced in N, N + NNG, and N + E compared to control. However, rats in N + NNG + E group performed a locomotor activity near to that of control group. In additions, nicotine significantly (*p* < 0.05) decreased the freezing time and immobility at day 15 of nicotine injection. However, NNG, N + NNG, N + E and N + NNG + E significantly (*p* < 0.05) prolonged the freezing time of rats at day 15 of nicotine injection. Moreover, N + NNG significantly (*p* < 0.05) increased the frequency and duration of rat's entrance into central squares at days 15 and 30 while N + NNG + E increased them significantly (*p* < 0.05) at day 15 only. Thus, the administration of nicotine-treated rats with NNG either alone in N + NNG group or in combination with vitamin E in N + NNG + E group could improve rats^,^ exploratory behaviors.

Table 2 illustrates that the working memory (SAP) was significantly (*p* < 0.05) decreased in N, N + NNG, and N + E groups, while it was moderately improved in the combination (N + NNG + E) group. DI was significantly (*p* < 0.05) decreased in the N group. The prophylactic administration of vitamin E and/or combination treatment with NNG and vitamin E showed an ameliorative effect on cognition by the restoration of the values to near normalcy.

### 3.2. Biochemical Changes

**Figure 2:**
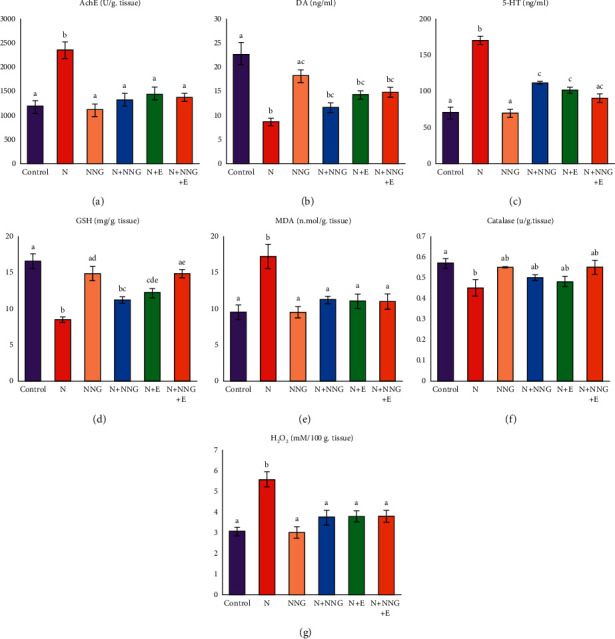
Effect of different treatments on (a) acetylcholinesterase activity, (b) dopamine (DA), (c) serotonin (5-HT), (d) GSH, (e) MDA, (f) catalase, and (g) H_2_O_2_ concentrations in the brain tissue of rats in different groups. Results are expressed as means ± SE (*n* = 10). Values with different letters in a column are significantly different at level *p* < 0.05. N: nicotine, NNG: nanonaringenin, E: vitamin E, DA: dopamine, 5-HT: serotonin, GSH: reduced glutathione, MDA: malondialdehyde, H_2_O_2_: hydrogen peroxide.

Figure 2 shows the activity of the AchE enzyme and the concentrations of dopamine and serotonin in brain tissue homogenates. AchE activity and serotonin content significantly increased (*p* < 0.05) in brain tissue of nicotine-treated rats compared with those in the control group. However, the concentration of dopamine significantly reduced (*p* < 0.05) in the nicotine group in comparison to the control group. Figure 2 also shows that the GSH concentration and catalase activity significantly decreased while the concentrations of MDA and H_2_O_2_ significantly increased (*p* < 0.05) in brain tissue homogenates of nicotine-treated rats compared with those in the control group. On the other hand, the prophylactic treatment with NNG and/or vitamin E parallel with nicotine administration showed an ameliorative effect by the restoration of these biomarkers' values to near normalcy. The supplementation of the nicotine-treated rats with either NNG or vitamin E induced the same degree of protection. In addition, NNG supplementation to normal rats did not alter these values in comparison to those in the control group, indicating no adverse effect of NNG on the brain.

### 3.3. Histological Changes of the Brain Tissue

**Figure 3:**
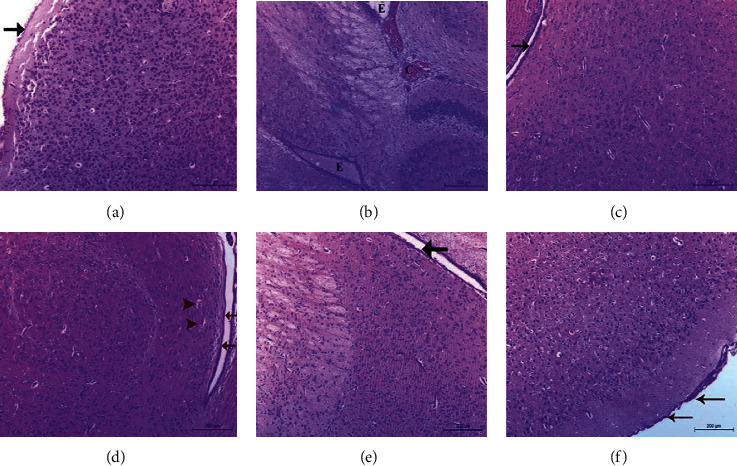
A photomicrograph of brain tissue in adult male albino rats showing the groups. (a) Control group, the brain tissue was covered by well-demarcated normal fine fibrous meninges (arrow). The brain tissue appeared normal in constituent with normal nerve cells and glial tissue. The meningeal and cerebral blood vessels appeared normal. (b) N group, the brain tissue covered with meninges suffered from thickening, congestion of meningeal blood vessels (C), and submeningeal edema (E). Both nerve cells and glial tissue showed degenerative changes. (c) NNG group, the brain tissue, and the meningeal layers (arrow) appeared normal. (d) N + E group, the brain tissue, and meninges (arrow) appeared normal. The cortical blood vessels suffered from congestion (arrow head). (e) N + NNG group, the brain tissue covered with normal meninges (arrow). The nerve cells and glial tissue appeared normal. (f) N + NNG + E group, the meningeal layers (arrow), and the brain tissue appeared normal. H&E stain X 100.

**Figure 4:**
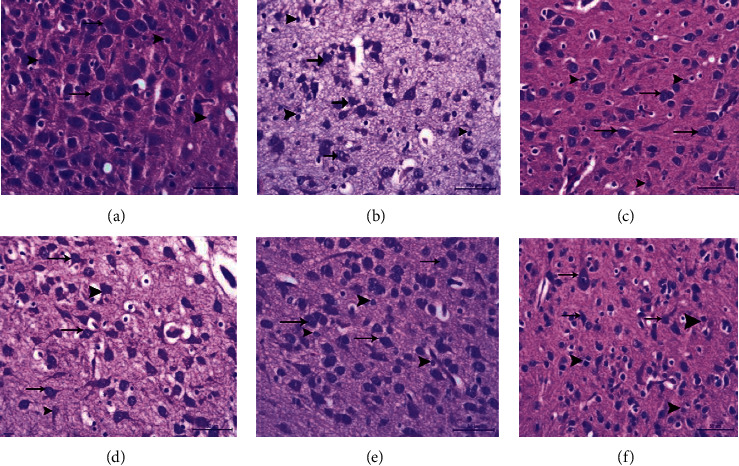
A higher magnification of brain tissue in adult male albino rats showing the groups. (a) Control group, showing normal nerve cells with a large spherical vesicular nucleus and basophilic cytoplasm (arrow). The neuroglia cells (arrow head) appeared normal and adjacent to the neurons. (b) N group, the majority of nerve cells appeared degenerated (arrow) the neuroglia cells (arrow head) appeared suffering from degeneration and necrosis. (c) NNG group, both nerve cells (arrow) and neuroglia (arrow head) appeared normal. (d) N + E group, nerve cells appeared normal (arrow) while other neurons suffered from degeneration (arrow head). (e) N + NNG group, the majority of neurons seemed to be normal (arrow) and the neuroglia cells appeared normal around neurons. (f) N + NNG + E group, the neurons (arrow) and the neuroglia cells appeared normal. H&E stain X 400.

**Figure 5:**
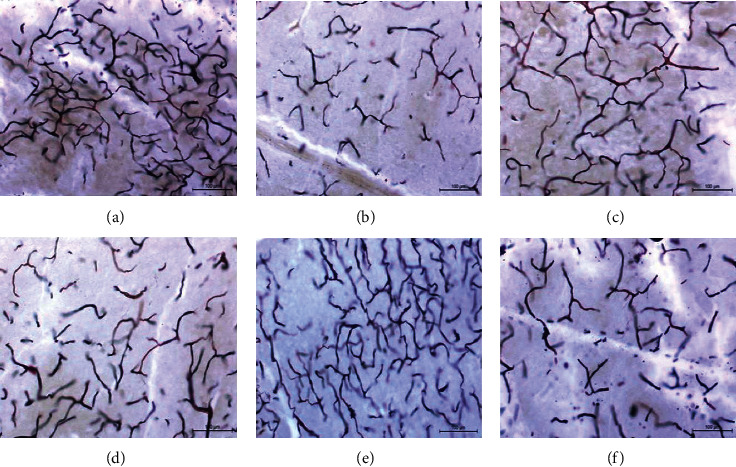
A histological picture of brain tissue in adult male albino rats showing the groups. (a) Control group, showing long and highly branched nerve fibers. (b) N group, the nerve fibers appeared short and less branched. (c) NNG group, the nerve fibers appeared long and highly branched. (d) N + E group, showing few nerve fibers appeared long and branched while the other nerve fibers appeared short and less branched. (e) N + NNG group, showing the majority of nerve fibers appeared long and highly branched. (f) N + NNG + E group, showing the nerve fibers appeared long and highly branched. Silver stain X 200.

The influence of different treatments on the nicotine-induced histopathological alterations in brain tissue is presented in Figures 3–5. Nicotine administration caused a thickening and congestion of the meninges and degeneration of the cerebral neurons and glia cells. The nerve fibers appeared short and less branched. NNG and/or vitamin E exhibited an improvement in brain structure.

## 4. Discussion

Nicotine stimulates the release of different neurotransmitters such as acetylcholine, dopamine, norepinephrine, and serotonin (5-HT) by nAChRs activation [3]. Nicotine effect on rat's behavior media by stimulation and desensitization of nAChRs is varied following acute and chronic nicotine administration [59]. The obtained data in our study indicated that nicotine might induce both rat's hypoactivity and hyperactivity. Nicotine was reported to reduce or increase activity (locomotion and rearing) in rats [60]. Repeated intermittent injections of nicotine may cause an increase in locomotor activity [61]. However, activity sensitization was not observed after continuous nicotine exposure [62]. According to Domino [63], the first dose of nicotine (0.32 mg/kg) could induce marked hypoactivity. Hence, the effect of nicotine on locomotion depends on sex [64], dose, and duration of exposure.

Monoamines are an important group of neuromodulators that are released onto spinal cord circuits and are critical for the expression of locomotion [65]. Dopamine plays a central role in the stimulation and modulation of the motor system in vertebrates and invertebrates [65, 66]. Animals with low DA levels exhibited hypoactivity and had impaired learning capability [67]. Thus, the recorded alteration in rats' activity is owing to the reported brain monoamines changes in our study.

The obtained results showed that the NNG and vitamin E did not ameliorate the prominent reduction in rat's activity caused by nicotine at 15 days of the experiment, while their combination successfully relieved the nicotine effect on locomotion at day 30. This indicated that the ameliorative effect of vitamin E [68, 69] and NNG became more potent at 30 days. Therefore, vitamin E and NNG combination is suggested in long-term nicotine administration.

Interestingly, NNG, vitamin E, and their combination prolonged immobility time at 15 days of the experiment. Tobacco was recorded to decrease vitamins C and E [68, 69]. In addition, adverse effects of some antioxidant supplementation on animal and human exercise performance were reported [70–72]. Hence, we hypothesize that the reported hypoactivity in N + NNG and N + E groups may be due to the high doses of their supplementation that were not decreased by the nicotine effect during the first 15 days of its administration.

The time rats spent freezing in the open field maze is an indicator of animal anxiety [44, 45]. In addition, the locomotion and rearing frequency of rats in a novel area are used to investigate their anxiety [44]. Less anxious rats in the novel area spent short freezing time and exhibited frequent motility such as rearing.

The observed behaviors declared that nicotine was anxiolytic on day 15 (decreased freezing time) and anxiogenic (decreased rearing) on day 30 of the experiment. These results are supported by early reports [3, 5, 73, 74] provided that nicotine had different effects on anxiety in both humans and animals depending on the species, strain, doses, route of administration, and experimental model used [5, 74, 75]. Acute injection of nicotine (0.01 mg/kg) was anxiogenic 5 min after injection and anxiolytic after 30 min [76]. Acute nicotine (0.35 mg/kg) injection had anxiogenic action [77] while chronic (15 days, 0.35 mg/kg) nicotine administration induced anxiolytic effect on the rat [77, 78]. In our experiment, the anxiogenic effect of nicotine on day 30 may be owing to the recorded decrease in brain DA [79].

Our data revealed that NNG could ameliorate the anxiogenic effect of nicotine. Similarly, NNG alleviated anxiety impairment induced by deltamethrin in rats [80]. This may be attributed to the capability of NNG to decrease AchE activities in addition to its neuroprotective and antistress effect [81].

The obtained results showed that the administration of NNG alone or in combination with vitamin E could successfully mitigate the nicotine effect on activity. Meanwhile, NNG improved the exploratory behavior of rats. Likewise, NNG treated rats exhibited a long exploration time [80]. Exploration is an indication of anxiety. Hence, NNG and vitamin E successfully decreased anxiety in treated rats. This may be due to the efficacy of NNG to decrease the anxiety that causes an increase in exploratory behavior and improves memory [82].

Our findings indicated that the tested dose of nicotine impaired the working memory and cognition of rats. In the early studies, nicotine caused impairments in learning and memory [83]. In additions, heavy smoking led to reduced cognitive function evaluated in mid-life [84]. On the other hand, nicotine enhanced cognitive function in experimental animals [85] or had no effect [86] on memory. Moreover, nicotine was found to improve learning at low doses, while higher doses impaired spatial memory in experimental animals [87]. Hence, the effect of nicotine on learning and memory seemed to depend critically upon some factors such as the test conducted, the administered dose of the nicotine, and the route of administration. In fact, the hippocampus had a significant role in learning and memory, especially spatial memory [88]. Also, intermittent injection of nicotine enhances nicotinic receptors and stimulates the mesolimbic and nigrostriatal dopaminergic pathways and the noradrenergic projections to the hippocampus, whereas continuous nicotine administration inhibits these responses [89]. Thus, we hypothesize that the deficits in spatial memory detected in the current study may be due to neuronal nicotinic receptors desensitization or the reported degenerative changes in cortical nerve cells and glial tissue. Furthermore, acetylcholine is one of the brain neurotransmitters included in cognitive and attention processes [90]. Thus, reported memory impairments may be attributed to the detected decrease in acetylcholine levels induced by the recorded increased brain AchE activity in our study.

Our data suggested that vitamin E had an ameliorative effect on the impaired cognition induced by nicotine. Meanwhile, a combination of vitamin E and NNG improved the cognition capability of rats. NNG could improve learning in experimental animals [25, 91]. Vitamin E and C administration in a small dose reduced memory deficit in APP/PSEN1 mice and improved performance of wild-type mice in the water maze while a higher dose of vitamin E with C had little decreasing oxidative stress effect than vitamin C only or the little dose of vitamins E and C, however, a combination of a great dose also weakened water maze performance in mice of both genotypes [92]. NNG has successfully ameliorated learning deficits mediated by inhibition of brain AchE activity [26] because it has an antiapoptotic effect [93]. Vitamin E alone was reported to attenuate the effect of hypoxia on the learning of rats [94, 95] and improved learning memory deficit [96]. On the contrary, no improvement or impairments in cognition were found following the administration of vitamin E [97–99]. Vitamin E ameliorated learning deficits induced by nicotine might be due to its antioxidant effect in the central nervous system [100].

The reported changes in brain monoamines (decrease DA and increase 5-HT) levels are consistent with Perez et al. [101], who found that prolonged nicotine treatment reduces the liberation of DA. Shearman et al. [102] reported that nicotine might induce a decrease or increase in DA and 5-HT levels. 5-HT was increased in response to high nicotine (0.5 mg/kg) doses while it decreased at low concentration (0.03 mg/kg). In addition, the reported increase in brain AchE is consistent with Saad et al. [103], who found that nicotine (1 mg/kg) increased brain AchE levels in the rat.

We suggest six explanations for decreased brain DA in the present study. The first is that prolonged exposure to nicotine (LTN) leads to increase nAChR desensitization that may alter the response of DA terminals to distinct firing patterns [101]. The second is the decreased endogenous acetylcholine levels induced by LTN treatment, which might decrease electrically evoked DA release [101, 104]. The third is LTN injection may induced alterations in the mesocorticolimbic pathway that may modify the dopaminergic terminals (in nucleus accumbens) response to an electrical stimulus. Indeed, nicotine treatment regulates mesolimbic *α*4*β*2 nAChRs on GABAergic neurons (in the ventral tegmental area; VTA), which in turn leads to an increase in their firing and decreases dopaminergic activity [105]. The altered GABAergic and glutamatergic activity in VTA may reduce dopaminergic activity in the nucleus [106]. Fourth, LTN exposure may increase the action of dopamine D2 receptor inhibition of dopaminergic function directly or/by reducing the activity of cholinergic interneurons [107]. Fifth, nicotine increases levels of circulating corticosteroids in both animals and humans due to stimulation of the hypothalamic-pituitary adrenal axis [108]. Consequently, chronic stress attenuates DA release [109]. Sixth, nicotine encourages the synthesis and release of catecholamines (noradrenaline/adrenaline) from the adrenal medulla. Nicotine controls the expression of tyrosine hydroxylase from the adrenal medulla (which is the rate-limiting enzyme in catecholamine biosynthesis), dopamine hydroxylase (which changes dopamine to noradrenaline), and neuropeptide Y (which is coliberated with the catecholamines) [110]. In addition, exposure to nicotine prenatally raises blood noradrenaline levels at baseline [111].

The prophylactic treatment with NNG and/or vitamin E or a combination of both for 45 days showed an ameliorative effect as the values returned nearly to control. The supplementation of nicotine-treated rats with either NNG or vitamin E induced the same degree of protection. Similarly, Rahigude et al. [112] and Khajevand-Khazaei et al. [26] stated that naringenin inhibits brain AchE in the rat. Liaquat et al. [113] reported that administration of naringenin improved cholinergic neurotransmission, which was detected by inhibited AchE activity, and increased acetylcholine levels in the whole brain of flavonoid fed rats. This decreased AchE activity led to increasing acetylcholine levels in synapses which resulted in improving cholinergic neurotransmission and eventually improved cognitive functions [113]. Therefore, it is believed that drugs that can increase acetylcholine levels and promote cholinergic functions may have the capacity for the therapeutic management of cognitive dysfunction such as dementia and Alzheimer's disease. In addition, naringenin prompted neuroprotective activity against neurotoxicity caused by 6-hydroxydopamine [114] via activation of Nrf2 signaling.

Nicotine promotes oxidative stress, implicates in several brain dysfunction, and prompts neurodegeneration. Experimental animal exposure to nicotine provokes oxidative stress and histopathological alterations due to diminishing the structural integrity and function of the brain [115, 116]. Peroxidizable fatty acids, which are present abundantly in neuronal membranes, are highly vulnerable to lipid peroxidation [117], which finally leads to the liberation of oxidative markers such as MDA and 4-hydroxynonenal [9]. ROS overproduction successfully caused lipid peroxidation in brain tissues of nicotine-treated rats, which was detected in the current study by a significant increase in MDA and H_2_O_2_ concentrations that, in turn, utilize GSH and catalase enzyme resulting in a significant decrease in GSH content and catalase activity in the brain tissue of nicotine-treated group compared to the control group (Figure 2). This data suggests that nicotine may exert its toxic action via redox imbalance. Similarly, Budzynska et al. [118] found that nicotine promoted lipid peroxidation in the brain tissues, which may have a major role in the development of brain dysfunction. The observed nicotine-induced significant depletion of brain GSH levels in the current study may indicate the decreasing of GSH synthesis and the more consumption of GSH for counteracting the nicotine-induced oxidative stress. Catalase catalyzes the breakdown of H_2_O_2_ into water and oxygen. The reduced tissue catalase activity detected in the current investigation may be owing to the increased ROS generation and the accumulation of superoxide radicals and H_2_O_2_ and, consequently, the increased utilization of these antioxidants to counter lipid peroxidation [118, 119]. In matching with our data, previous studies had reported similar results of nicotine toxicity in different rat tissues [120, 121]. Besides, Helen et al. [122] observed an increase in hydroperoxide levels in the lung, liver, and kidney of nicotine-administered rats. Cognitive impairment occurs due to reduced activity of the antioxidant defense system [113, 123].

Pre- and cotreatments of nicotine-treated rats with either NNG and/or vitamin E significantly raised the levels of antioxidants like GSH and catalase enzyme but significantly reduced MDA and H_2_O_2_ concentrations. This action was augmented by the histopathological picture of the brain tissue, especially neurons and neuroglia cells (Figures 3–5). Interestingly, the degree of improvement in MDA and GSH levels was markedly higher in the combination group reflecting the synergistic antioxidant effects of both treatments by augmenting antioxidant defense mechanisms than the single use. In this group, neurons appeared normal without any degree of impairment and the nerve fibers became highly branched.

Vitamin E, the major lipophilic antioxidant, plays an important role in the protection against oxidative stress and protects cell membranes from oxidative damage [124]. It is demonstrated that vitamin E can also protect against lipid peroxidation caused by nicotine in animals [125, 126] and humans [127, 128], therefore preventing lipid peroxidation and raising the antioxidant status in nicotine-treated rats [129, 130]. Vitamin E prevented nicotine-induced lipid peroxidation and GSH depletion in brain tissue, as it might have easily diffused to rat brain as a lipid-soluble antioxidant [131].

Several studies reported that flavonoids can cross Blood-brain barrier (BBB), where they act as potent antioxidants and protect nerve cells against oxidative damage [132, 133]. Naringenin is a well-known flavonoid abundant in citrus fruits [134]. The lipophilic nature of naringenin favors a good BBB-permeability [135, 136]. Naringenin uptake into the cerebral cortex and the striatum [137, 138] proposes that naringenin should allow protection of the neurons throughout the CNS. A previous study had postulated that naringenin could enhance learning and memory retention by improving antioxidant enzyme activities in rats [113]. Other reports documented that oral supplementation of naringenin significantly enhanced antioxidant enzyme activities [139, 140]. In our study, TEM analysis revealed that the prepared NNG possesses a size up to 100 nm with a spherical shape indicative of better uptake of nanoparticles by cells. Naringenin antioxidant properties have been earlier described [112]. Naringenin antioxidant properties are related to its potential scavenging effect on both O^2−^, OH^−^, and lipid peroxide owing to the presence of hydroxyl groups in its structure, which possesses electron-donating properties, thereby protecting membranes from the free radical attack [141].

As an anticholinesterase, NNG enhances memory inefficiency in diabetes [112] and cognitive deficit induced by intracerebroventricular-streptozotocin [139]. Other studies recommended that pretreatment with naringenin promotes locomotion and raised glutathione with reduced MDA content in the brain tissue of Parkinson's disease (PD) rat model [142]. All these findings indicated that NNG can be considered as a potential neuroprotective agent and improve learning and memory via its antioxidant activity.

In the current study, the histopathological examinations support the biochemical analysis where the nicotine caused marked pathological changes in the cerebral cortex indicated by structural alterations, congestion of meningeal and cerebral blood vessels, prevascular edema, and degeneration of the cortical nerve cells and glial tissue. Cotreatment with oral NNG and/or vitamin E attenuates these changes in cerebral structure. Structural observations have confirmed the cerebroprotection nature of NNG and/or vitamin E on nicotine-induced oxidative lesions.

## 5. Conclusion

Our findings suggested that nicotine may lead to oxidative stress and impairment in behavioral, biochemical, and histological variables. However, supplementation of nanonaringenin (NNG), vitamin E, and their combination for 45 days might exert successful amelioration of theses impairments as well as brain AchE, monoamines and redox markers alterations. Thus, NNG and vitamin E should be included in the diet to afford a protective effect against nicotine-induced cytotoxicity. Further studies are required to evaluate the efficacy of different doses of NNG and vitamin E to mitigate impairments induced by nicotine at different routes of administration and durations.

## Figures and Tables

**Table 1:** Effect of treatments on locomotor and exploratory behavior of rats (in the open field test).

	Locomotor and anxiety like behaviors						Exploratory behaviors			
	No. of peripheral square crosses		Rearing (frequency)		Freezing duration (sec)		No. of central square crosses		Central square time (sec)	
	15 days	30 days	15 days	30 days	15 days	30 days	15 days	30 days	15 days	30 days
Control	67.4 ± 2.79 ^ac^	38.2 ± 9.65^a^	12.0 ± 1.58^a^	8.4 ± 2.07^a^	61.8 ± 6.49^a^	119 ± 44.22^a^	2.4 ± 1.34^a^	1.8 ± 0.45^a^	3.0 ± 2.1^a^	1.6 ± 0.89^a^
N	27.2 ± 5.63^b^	16.8 ± 3.96^b^	16.8 ± 3.3^b^	2.4 ± 1.14^b^	28.6 ± 3.21^b^	103.2 ± 16.16^a^	1.4 ± 0.55^a^	1.4 ± 0.55^a^	2.4 ± 1.1^a^	2.4 ± 0.86^a^
NNG	74.4 ± 4.7^a^	36.4 ± 2.7^a^	9.8 ± 0.84 ^ad^	8.52 ± 1.41^a^	139.6 ± 8.26^c^	114.6 ± 12.68^a^	2.8 ± 1.92^a^	1.2 ± 0.84^a^	4.0 ± 1.2 ^ab^	1.0 ± 1.0^a^
N + NNG	39.2 ± 9.5 ^bd^	30.8 ± 4.76^a^	6.6 ± 0.89 ^cd^	3.2 ± 2.17^b^	111 ± 23.01^c^	122 ± 28.64^a^	6.6 ± 2.88^b^	5.2 ± 1.8^b^	7.6 ± 1.52^b^	7.2 ± 1.9^b^
N + E	46.4 ± 19.8 ^cb^	16.0 ± 4.69^b^	5.4 ± 1.14^c^	2.0 ± 1.58^b^	119 ± 18.84^c^	138 ± 28.64^a^	2.4 ± 2.70^a^	1.4 ± 0.55^a^	3.0 ± 1.58^a^	1.6 ± 0.89^a^
N + NNG + E	50.4 ± 15.6 ^cd^	37.2 ± 7.56^a^	6.8 ± 1.3 ^cd^	10.0 ± 1.23^a^	236 ± 23.02^d^	130 ± 30.82^a^	4.8 ± 0.45 ^ab^	1.6 ± 1.52^a^	7.4 ± 3.36^b^	1.8 ± 0.84^a^

**Table 2:** Effect of treatments on learning and cognition of rats.

	Spontaneous alternation behavior percent (SAP)	Novel object recognition discrimination index (DI)
Control	33.8 ± 2.17^a^	0.406 ± 0.10^ab^
N	28.2 ± 3.03^bc^	0.288 ± 0.08^b^
NNG	32.0 ± 3.39^ab^	0.464 ± 0.07^a^
N + NNG	28.2 ± 1.79^bc^	0.396 ± 0.07^ab^
N + E	26.6 ± 3.21^c^	0.536 ± 0.07^a^
N + NNG + E	31.2 ± 2.39^abc^	0.456 ± 0.06^a^

## Data Availability

All data used to support the findings of this study are available from the corresponding author upon request.
